# Effect of Low Versus High Tidal-Volume Total Liquid Ventilation on Pulmonary Inflammation

**DOI:** 10.3389/fphys.2020.00603

**Published:** 2020-06-18

**Authors:** Michaël Sage, Wendy See, Stéphanie Nault, Christophe Morin, Christina Michalski, Benoit Chabot, Sofia Marouan, Pascal M. Lavoie, Philippe Micheau, Jean-Paul Praud, Étienne Fortin-Pellerin

**Affiliations:** ^1^Department of Pharmacology-Physiology, Université de Sherbrooke, Sherbrooke, QC, Canada; ^2^BC Children’s Hospital Research Institute, The University of British Columbia, Vancouver, BC, Canada; ^3^Department of Experimental Medicine, The University of British Columbia, Vancouver, BC, Canada; ^4^Department of Microbiology and Infectiology, Université de Sherbrooke, Sherbrooke, QC, Canada; ^5^Department of Pathology, Université de Sherbrooke, Sherbrooke, QC, Canada; ^6^Department of Pediatrics, The University of British Columbia, Vancouver, BC, Canada; ^7^Department of Mechanical Engineering, Université de Sherbrooke, Sherbrooke, QC, Canada; ^8^Department of Pediatrics, Université de Sherbrooke, Sherbrooke, QC, Canada

**Keywords:** liquid ventilation, ventilator-induced lung injury, tidal volume, newborn lamb, transcriptome

## Abstract

Animal experiments suggest that total liquid ventilation (TLV) induces less ventilator-induced lung injury (VILI) than conventional mechanical gas ventilation. However, TLV parameters that optimally minimize VILI in newborns remain unknown. Our objective was to compare lung inflammation between low (L-V_T_) and high (H-V_T_) liquid tidal volume and evaluate impacts on the weaning process. Sixteen anesthetized and paralyzed newborn lambs were randomized in an L-V_T_ group (initial tidal volume of 10 mL/kg at 10/min) and an H-V_T_ group (initial tidal volume of 20 mL/kg at 5/min). Five unventilated newborn lambs served as controls. After 4 h of TLV in the supine position, the lambs were weaned in the prone position for another 4 h. The levels of respiratory support needed during the 4 h post-TLV were compared. The anterior and posterior lung regions were assessed by a histological score and real-time quantitative PCR for *IL1B*, *IL6*, and *TNF* plus 12 other exploratory VILI-associated genes. All but one lamb were successfully extubated within 2 h post-TLV (72 ± 26 min vs. 63 ± 25 min, *p* = 0.5) with similar FiO_2_ at 4 h post-TLV (27 ± 6% vs. 33 ± 7%, *p* = 0.3) between the L-V_T_ and H-V_T_ lambs. No significant differences were measured in histological inflammation scores between L-V_T_ and H-V_T_ lambs, although lambs in both groups exhibited slightly higher scores than the control lambs. The L-V_T_ group displayed higher *IL1B* mRNA expression than the H-V_T_ group in both anterior (2.8 ± 1.5-fold increase vs. 1.3 ± 0.4-fold increase, *p* = 0.02) and posterior lung regions (3.0 ± 1.0-fold change increase vs. 1.1 ± 0.3-fold increase, *p* = 0.002), respectively. No significant differences were found in *IL6* and *TNF* expression levels. Gene expression changes overall indicated that L-V_T_ was associated with a qualitatively distinct inflammatory gene expression profiles compared to H-V_T_, which may indicate different clinical effects. In light of these findings, further mechanistic studies are warranted. In conclusion, we found no advantage of lower tidal volume use, which was in fact associated with a slightly unfavorable pattern of inflammatory gene expression.

## Introduction

Despite major advances in care, respiratory support remains challenging for many infants hospitalized in the neonatal intensive care unit. Total liquid ventilation has been suggested as an alternative to conventional mechanical ventilation for various conditions affecting the neonate, such as children born with diaphragmatic hernia (Snoek et al., 2014), meconium aspiration syndrome (Avoine et al., 2011) or who are born extremely premature (Shaffer et al., 1999; Davidson and Berkelhamer, 2017). Total liquid ventilation (TLV) uses liquid perfluorochemicals (PFCs), such as perflubron (PFOB, perfluorooctyl bromide), together with a dedicated liquid ventilator. In comparison to conventional gas ventilation, this was shown to ameliorate tidal-volume distribution and to require less positive pressure, thus reducing VILI in animal models (Tooley et al., 1996; Wolfson et al., 2008; Avoine et al., 2011; Pohlmann et al., 2011; Sage et al., 2018b). Perflubron, the PFC used for this study, also has anti-inflammatory properties that could reduce VILI (Thomassen et al., 1997; Woods et al., 2000).

Over the past decade, our team has developed a liquid ventilator prototype that allows for precise control over pressures and flows delivered to the lungs (Robert et al., 2010; Sage et al., 2018a, b). These advances have allowed for tighter control over the end-expiratory lung volume of PFC, mainly through prevention of tracheal collapse occurring when excessive negative pressure is applied during expiration. These repetitive tracheal collapses cause progressive accumulation and trapping of fluid in the lungs (Costantino et al., 2004; Bagnoli et al., 2007). Our most recent prototype, called INOLIVENT, can be used to refine ventilation algorithms and lung protective strategies during TLV (Nadeau et al., 2018; Sage et al., 2018a; Kohlhauer et al., 2019).

High tidal volumes with conventional gas ventilation have been especially cited as inducing greater VILI (Ciuffini et al., 2018; Farrell et al., 2018). Our ventilation algorithms allow higher respiratory rates and lower tidal volumes to be used, thus maintaining minute ventilation, while avoiding tracheal collapses (up to ∼10 cycles/min compared to 3–6 cycles/min with previously published TLV prototypes) (Robert et al., 2007, 2009, 2010). The higher proportion of liquid tidal volume cycling within the anatomic dead space is, however, of particular concern in the context of low gas diffusion in PFC (Koen et al., 1988; Costantino and Fiore, 2001). To our knowledge, the effect of tidal volume and respiratory rate on lung inflammation during TLV has not been thoroughly addressed. Jiang et al. (2016) showed that a tidal volume of 6 mL/kg was associated with lower transcription and plasma protein levels of IL-6 and IL-8 in piglets, compared to a higher tidal volume of 25 mL/kg during TLV (Jiang et al., 2016). The authors, however, had to use extracorporeal carbon dioxide removal to achieve acceptable blood-gas exchange, thus preventing the need for high minute ventilation and its potential impact on VILI occurrence. Extracorporeal carbon dioxide removal is associated with serious adverse effects and is not currently an option for extremely low birth-weight neonates (Liu et al., 2016). Kohlhauer et al. (2019) recently demonstrated that using low intrapulmonary PFOB volumes, measured as the amount of breathable liquid in the lung at the end expiratory pause (EELV), during a short 30-minute course of TLV was associated with less pulmonary inflammation. The main objective of the present study was to compare lung inflammation induced by low vs. high tidal-volume TLV in a healthy term lamb model without extracorporeal carbon dioxide removal, while maintaining sustainable gas exchanges for a prolonged period. We hypothesize that, like conventional mechanical gas ventilation, high tidal volume (H-V_T_) will result in greater lung inflammation and impede complete post-TLV weaning compared to low tidal volume (L-V_T_).

## Materials and Methods

### Lamb Instrumentation

A total of 21 full-term healthy newborn male lambs aged 3.3 ± 1.2 days were brought to the research facility from a local breeder. The study was approved by the animal research ethics board of the Université de Sherbrooke (protocol #417-17) and performed in accordance with the Canadian Council on Animal Care guidelines.

Sixteen lambs subjected to TLV were premedicated with intramuscular ketamine (10 mg/kg) prior to percutaneous cannulation of the left jugular vein. They were then intubated with a 4.5 mm cuffed endotracheal tube and placed in the supine position. Gas ventilation was initiated (Servo-i, Maquet Critical Care, Solna, Sweden) in the pressure-controlled mode with a respiratory rate of 40 cycles/min and a positive end-expiratory pressure of 5 cmH_2_O. The inspiratory pressure was manually adjusted to deliver a tidal volume of approximately 7 mL/kg. Hemoglobin oxygen saturation was continuously recorded by pulse oximetry (Radical, Masimo, Irvine, CA, United States) with a probe at the base of the tail. Lambs were maintained under general anesthesia throughout the TLV phase of the experiment using an intravenous infusion of propofol 120 μg/kg/min and ketamine 1 mg/kg/h, together with a 10% dextrose solution at a rate of 5 mL/kg/h. A catheter was inserted into the left carotid artery under sterile conditions to allow for continuous monitoring of systemic arterial pressure and blood-gas measurements. Lambs were then randomized to either the L-V_T_ (high respiratory rate) or H-V_T_ (low respiratory rate) TLV group.

### Experimental Protocol

#### Total Liquid Ventilation

After surgical instrumentation under gas ventilation, the lambs were paralyzed (rocuronium bromide 0.2 mg/kg IV) and disconnected from the gas ventilator for 5 s to allow for lung deflation prior to TLV initiation. The lungs were then filled with 20 mL/kg of pre-oxygenated perfluorooctyl bromide (Exfluor, Round Rock, TX, United States) at 39°C over 18 s, using the INOLIVENT-6 liquid ventilator prototype as previously described (Sage et al., 2018a). Volume-controlled, pressure-limited, and time-cycled TLV was then initiated with a respiratory rate of 10 cycles/min and a tidal volume of 10 mL/kg (L-V_T_ group) or a respiratory rate of 5 cycles/min and a tidal volume of 20 mL/kg (H-V_T_ group). The inspiratory to expiratory time ratio was set at 1:2. The end-expiratory lung volume was progressively increased to 30 mL/kg over the first 10 respiratory cycles to reach a lung volume similar to functional residual capacity (25–30 mL/kg) during conventional mechanical ventilation in this model (Overfield et al., 2001; Wolfson and Shaffer, 2004). Given the complexity of changing respiratory rate with our current liquid ventilator algorithms, respiratory rate was kept constant throughout TLV and tidal volumes were adjusted to maintain targeted blood gases using a permissive hypercapnia approach (pH ≥ 7.20 and PaCO_2_ from 50 to 65 mmHg (Ryu et al., 2012). Oxygen concentration in the PFC was adjusted in order to maintain pulse oximetry values above 90%. After 4 h of total liquid ventilation, the lambs were placed in the prone position. Neostigmine (0.05 mg/kg) and glycopyrrolate (0.01 mg/kg) were administered to reverse rocuronium effect, and the anesthetic infusion was stopped, allowing the lambs to recover from anesthesia. TLV was halted after an expiration, lambs were disconnected from the liquid ventilator and placed in decline position to favor liquid draining into a bottle by gravity. When no more PFOB was coming out of the endotracheal tube (≈10 s), the gas ventilator was connected.

#### Weaning From Tidal Liquid Ventilation

**FIGURE 1:**
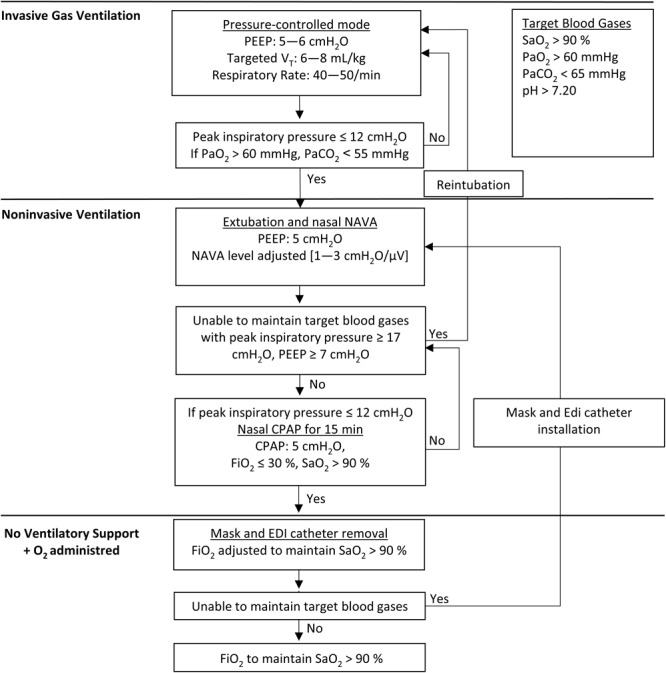
Standardized weaning protocol after total liquid ventilation. PaCO_2_: partial pressure of carbon dioxide in arterial blood, PaO_2_: partial pressure of oxygen in arterial blood, SaO_2_: hemoglobin saturation in oxygen (pulse oximetry), PEEP: positive end-expiratory pressure, VT: tidal volume. CPAP: continuous positive airway pressure, NAVA: neurally adjusted ventilatory assist.

Figure 1 shows the standardized weaning protocol used. After TLV, the animals were weaned to conventional gas ventilation in the pressure-controlled mode. The initial FiO_2_ and ventilatory parameters (peak inspiratory pressure of 15 cmH_2_O, PEEP of 6 cmH_2_O, respiratory rate of 40/min) were adjusted throughout the weaning protocol to maintain tidal volume at 6–8 mL/kg, SaO_2_ > 90%, and PaO_2_ between 60 and 80 mmHg, PaCO_2_ between 50 and 65 mmHg, and pH > 7.20. A nasal catheter was inserted into the esophagus to record the electrical activity of the diaphragm (Edi) (Maquet Critical Care, Solna, Sweden) and a nasal mask custom-made for lambs was installed. The lambs were then extubated as soon as they had spontaneous, regular breathing movements with peak inspiratory pressure less than or equal to 12 cmH_2_O to generate adequate tidal volumes and placed under nasal neurally adjusted ventilatory assist (NAVA). The PEEP, FiO_2_ and NAVA levels were adjusted to maintain SaO_2_ above 90% and to minimize the clinically assessed work of breathing. The NAVA level was thereafter decreased until the lambs could be switched to nasal CPAP at 5 cmH_2_O for at least 15 min. The mask was then removed, and supplemental oxygen was delivered via nasal cannulae to maintain SaO_2_ >90% and PaO_2_ between 60 and 80 mmHg. If PaCO_2_ was above 65 mmHg at any point, the nasal CPAP at 5 cmH_2_O was reinstalled. If needed, nasal pressure support ventilation and even endotracheal pressure-controlled ventilation could have been instituted again based on the same criteria. The lambs were euthanized 4 h post-TLV with an IV injection of 90 mg/kg pentobarbital.

#### Control Group

Five lambs were used for histological and RNA transcript analysis without undergoing mechanical ventilation or any other experimental procedure. They were euthanized after peripheral venous cannulation and lung samples were collected.

#### Histological Score for Lung Inflammation

Samples (2 × 2 × 0.5 cm) from the same region of the peripheral anterior lower lobe and posterior lower lobe of the right lung were collected, fixed, and conserved in 10% formaldehyde. The tissues were then embedded in paraffin, from which 5 μm sections were prepared for hematoxylin and eosin staining. A blinded pathologist examined the slides for the experimental and control groups (SM) using a histological score of lung inflammation previously developed for the newborn lamb (Hillman et al., 2010). Four key components (septation thickness, hemorrhage, inflammatory-cell infiltration, and epithelial sloughing) were assessed, each on a scale of 0 to 2 (total inflammation score of 8).

#### Gene Transcription Analysis

Samples were collected in the left lung from the same regions as for the histological samples from the right lung and assayed for *IL1B*, *IL6*, and *TNF* gene transcription levels. As the focus of our laboratory will be the use of TLV to prevent bronchopulmonary dysplasia in the years to come, we have oriented the present exploratory analyses as such. We measured the transcription level of 12 other genes selected based on their implication in VILI and bronchopulmonary dysplasia development: *AGER, CCL4, CSF2, CXCL1, CXCL8, ICAM1, IL1A, IL1R1, IL33, NFKB1, NFKB2*, and *TNRRSF1A* (Moldoveanu et al., 2008; Huusko et al., 2014). To this end, total RNA extraction was performed on tissue samples preserved in TRIzol (Invitrogen, Carlsbad, CA, United States) at −80°C using TissueLyser (Qiagen, Toronto, ON, Canada). Chloroform was added following the manufacturer’s protocol. The aqueous layer was recovered, then mixed with one volume of 70% ethanol and applied directly to a RNeasy Mini Kit column (Qiagen). DNAse treatment and total RNA recovery were performed as per the manufacturer’s protocol. RNA quality and presence of contaminating genomic DNA were verified as previously described (Brosseau et al., 2010). RNA integrity was assessed with an Agilent 2100 Bioanalyzer (Agilent Technologies, Santa Clara, CA, United States). Reverse transcription was performed on 1.1 μg total RNA with Transcriptor reverse transcriptase, Random Hexamer Primers, deoxynucleoside triphosphate kit (Roche Diagnostics, Basel, Switzerland), and 10 units of RNAseOUT (Invitrogen) according to the manufacturer’s protocol in a total volume of 10 μL. Individual forward and reverse primers were resuspended at 20 to 100 μM stock solution in Tris-EDTA buffer (Integrated DNA Technologies, Inc., Coralville, IA, United States). They were thereafter diluted as a primer pair to 1 μM in RNase DNase-free water (Integrated DNA Technologies, Inc.). Quantitative PCR (qPCR) was performed in 384 well plates in a CFX-384 thermocycler (Bio-Rad, Hercules, CA, United States) with 5 μL of 2X iTaq Universal SYBR Green SuperMix (Bio-Rad), 10 ng (3 μL) cDNA, and 200 nM (2 μL) final primer pair solutions. The following cycling conditions were used: 3 min at 95°C, followed by 50 cycles (15 s at 95°C, 30 s at 60°C, and 30 s at 72°C). Relative expression levels were calculated with the qBase framework (Hellemans et al., 2007) and the housekeeping genes glyceraldehyde 3-phosphate dehydrogenase (GAPDH), Beta-2 microglobulin (B2M), ribosomal protein L13a (RPL13A), and TATA-binding protein (TBP) for lamb cDNA. Primer design and validation were evaluated as previously described (Brosseau et al., 2010). In every qPCR run, a no-template control was performed for each primer pair and the results were consistently negative. All primer sequences are available in Supplementary Table S1.

### Data Analysis

All RNA levels were normalized to a group of housekeeping-gene expression. RNA transcription results were presented in log_2_ (fold change between L-V_T_ and H-V_T_ lambs). Principal component analysis and hierarchical clustering were performed, as exploratory analyses, with R software^1^. Statistical analyses were performed with SPSS 19 (IBM, Armonk, NY, United States). Gene expression data in each group were reported as mean ± SD (unless specified otherwise), and the groups compared with a 2-sided non-paired *t*-test. Histological-score results were reported as median (Q1; Q3) and compared with the Kruskal–Wallis test followed by the Mann–Whitney *U* test. A value of *p* < 0.05 was deemed to be statistically significant.

## Results

The lambs from all groups were similar in age and weight (Table 1). All were successfully weaned to spontaneous breathing at 4 h post-TLV, except for one lamb in the H-V_T_ group that remained intubated because of anesthesia-related weak respiratory efforts; the lamb’s extubation time was not considered in the analysis.

**TABLE 1:** Lamb characteristics at baseline and blood gases.

**Parameters**	**ID**	**GV**	**TLV**		**Weaning**	
		**Baseline**	**2 h**	**4 h**	**6 h**	**8 h**
Age (Days of life)	L-V_T_	4.0 ± 1.2	–	–	–	–
	H-V_T_	3.0 ± 1.4	–	–	–	–
	CTL	2.8 ± 0.8	–	–	–	–
	*p value*	0.2	–	–	–	–
Weight (kg)	L-V_T_	3.8 ± 0.8	–	–	–	–
	H-V_T_	3.4 ± 0.6	–	–	–	–
	CTL	3.6 ± 0.4	–	–	–	–
	*p value*	0.2	–	–	–	–
PaO_2_ (mmHg)	L-V_T_	95 ± 13	108 ± 30	97 ± 32	86 ± 26^b^	75 ± 8^a,b^
	H-V_T_	82 ± 16	116 ± 34	104 ± 53	85 ± 19	90 ± 35
	*p value*	0.3	0.6	0.7	1.0	0.3
SaO_2_ (%)	L-V_T_	97 ± 3	99 ± 1	99 ± 2	96 ± 2	98 ± 2
	H-V_T_	97 ± 3	99 ± 1	99 ± 2	96 ± 2	98 ± 2
	*p value*	0.3	0.6	0.9	0.4	0.4
PaCO_2_ (mmHg)	L-V_T_	41 ± 5	62 ± 15^a^	60 ± 21^a^	41 ± 6^b,c^	39 ± 7^b,c^
	H-V_T_	38 ± 7	63 ± 12^a^	58 ± 17^a^	41 ± 7^b,c^	42 ± 5^b,c^
	*p value*	0.3	0.9	0.7	1.0	0.4
pH	L-V_T_	7.35 ± 0.07	7.21 ± 0.10^a^	7.26 ± 0.14	7.40 ± 0.07^a,c^	7.42 ± 0.06^a,b,c^
	H-V_T_	7.39 ± 0.10	7.22 ± 0.06^a^	7.23 ± 0.08	7.38 ± 0.07^b,c^	7.40 ± 0.05^b,c^
	*p value*	0.3	0.9	0.9	0.5	0.4
HC0_3_^–^ (mmol/L)	L-V_T_	21.3 ± 2.1	20.6 ± 3.0	21.4 ± 2.0	24.4 ± 3.2^a,c^	25.1 ± 3.6
	H-V_T_	21.9 ± 3.1	20.7 ± 1.3	20.3 ± 0.9	23.0 ± 2.3^C^	24.1+ 1.8^b,c^
	*p value*	0.7	0.9	0.2	0.3	0.5

*ID, Identification; GV, gas ventilation; TLV, total liquid ventilation; L-V_*T*,_ low tidal volume; H-V_*T*_: high tidal volume; CTL, control lambs without ventilation; PaO_2_, partial pressure of oxygen in arterial blood; SaO_2_, hemoglobin saturation in oxygen (pulse oximetry); PaCO_2_, partial pressure of carbon dioxide in arterial blood; HCO_3_^–^, arterial concentration of bicarbonate ions. *P* < 0.05 in comparison with ^*a*^: baseline; ^*b*^: 2 h time point; ^*c*^: 4 h time point.*

Table 2 provides the ventilatory parameters. They were identical in the H-V_T_ and L-V_T_ groups at all time points, except for tidal volume and respiratory rate. Tidal volume nevertheless needed to be raised in the L-V_T_ group from 10 mL/kg (at TLV initiation) to 14 ± 2 mL/kg within the first 2 h of TLV to achieve the targeted PaCO_2_/pH values; the tidal volume remained stable thereafter. Minute ventilation in the L-V_T_ group was thus significantly higher than in the H-V_T_ group in order to achieve similar PaCO_2_ and pH values, as expected. The end-expiratory lung volume of PFC was closely monitored and kept minimal in both groups, while avoiding end-expiratory tracheal collapse (35 ± 11 mL/kg).

**TABLE 2:** Ventilatory parameters.

**Parameters**	**ID**	**GV**	**TLV**		**Weaning**	
		**Baseline**	**2 h**	**4 h**	**6 h**	**8 h**
FiO_2_-FgO_2_ (%)	L-V_T_	276	9412	9312	317	276
	H-V_T_	245	966	968	3710	337
	*p* value	0.2	0.6	0.6	0.2	0.3
V_T_ (mL/kg)	L-V_T_	7.40.8	142	144	−	−
	H-V_T_	7.70.8	181	201^b^	−	−
	*p* value	0.5	<0.001	<0.001	−	−
Respiratory rate (/min)	L-V_T_	41 + 8	100	100	5812	626
	H-V_T_	447	50	50	6922	6012
	*p* value	0.4	<0.001	<0.001	0.2	0.7
V_*E*_ (mL/kg/min)	L-V_T_	30375	13722	14943	−	−
	H-V_T_	34163	907	1005^b^	−	−
	*p* value	0.3	<0.001	<0.001	−	−
PIP-EIPP (cmH_2_O)	L-V_T_	132	86	97^b^	−	−
	H-V_T_	13 + 4	93	93	−	−
	*p* value	0.3	0.5	0.8	−	−
PEEP - EEPP (cmH_2_O)	L-V_T_	61	04	04	−	−
	H-V_T_	50	−13	−24	−	−
	*p* value	0.1	0.5	0.3	−	−
EELV (mL/kg)	L-V_T_	−	325	3513	−	−
	H-V_T_	−	337	359	−	−
	*p* value	−	0.6	0.9	−	−

*FiO_2_, fraction of inspired oxygen; FgO_2_, fraction of oxygen bubbled in PFOB in the ventilator; V_*E*_, minute ventilation; PIP, peak inspiratory pressure; EIPP, end-inspiratory pause pressure; EELV, end-expiratory liquid volume; PEEP, positive end-expiratory pressure; EEPP, end-expiratory pause pressure (TLV). See legend of Table 1 for other abbreviations.*

Lambs in the L-V_T_ group were extubated after 72 ± 26 min, while those in the H-V_T_ group were extubated after 63 ± 25 min (*p* = 0.5). At 2 h post-TLV, four lambs in the L-V_T_ group and five lambs in the H-V_T_ group were under nasal NAVA. At 4 h post-TLV, one lamb in the L-V_T_ and two lambs in the H-V_T_ group remained under NAVA. The majority (seven in the L-V_T_, five in the H-V_T_ group) of the lambs were only on supplemental oxygen. FiO_2_ requirements at the end of the experiment were similar in the L-V_T_ and H-V_T_ groups at 27 ± 6 and 33 ± 7%, respectively, (*p* = 0.3). Blood gases were similar in both groups at all time points (Table 1).

### Histological Inflammation Score

**FIGURE 2:**
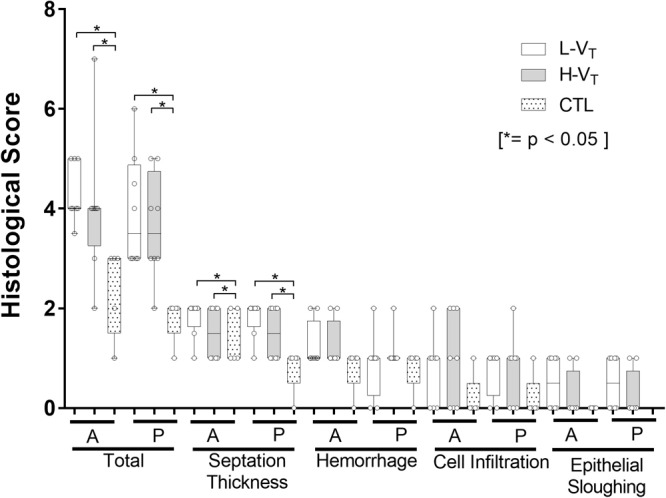
Inflammation histological score in lambs subjected to TLV with L-V_T_ (*n* = 8), H-V_T_ (*n* = 8), and CTL (*n* = 5). The total score out of eight represents the sum of the four categories scored over two (septation thickness, hemorrhage, cell infiltration, and epithelial sloughing). A: anterior region of the lung; P: posterior lung region. A higher score indicates more inflammation. Each point represents a lamb and each line represents the median and the first and third quartiles (Q1; Q3). *: statistically significant difference (*p* < 0.05).

Both TLV groups had significantly higher inflammation scores compared to the control non-ventilated group, mainly due to increased septation thickness (Figure 2). The histological score also tended to indicate higher inflammation in the L-V_T_ group compared to the H-V_T_ group in the anterior lung region, although this difference did not reach statistical significance (Figure 2).

### Inflammatory-Gene Expression

**FIGURE 3:**
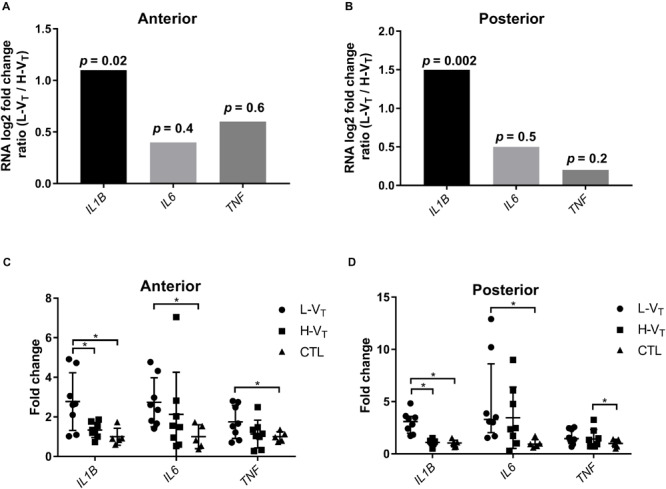
Expression of the *IL1B*, *IL6* and *TNF* genes illustrated by log_2_ fold changes in L-V_T_ relative to H-V_T_ lambs in the anterior **(A)** and posterior **(B)** lung regions, and fold changes in gene expression of L-V_T_, H-V_T_ and unventilated lambs relative to housekeeping genes using qBase relative quantification framework method [glyceraldehyde-3-phosphate dehydrogenase (GAPDH), beta-2-microglobulin (B2M), ribosomal protein L13a (RPL13A), and TATA-binding protein (TBP)] in the anterior **(C)** and posterior **(D)** lung regions. Each dot represents a lamb, with mean ± standard deviation. *p < 0.05. See legend of Table 1 for other abbreviations.

Lung tissue *IL1B* mRNA was significantly more expressed in the L-V_T_ group than the H-V_T_ group in both the anterior and posterior lung regions (Figures 3A,B). The expression of both *IL6* and *TNF* transcripts was greater in the L-V_T_ group, but the difference was not significant (Figures 3A,B). When compared to the control lambs, the L-V_T_ lambs exhibited a significantly higher expression of *IL1B* (*p* = 0.01), *IL6* (*p* = 0.006), and *TNF* (*p* = 0.04) in the anterior region, while *IL1B* and IL6 were significantly upregulated in the posterior region (*p* < 0.001; *p* = 0.03, respectively) (Figures 3C,D). On the other hand, only *TNF* was upregulated in the posterior region (*p* = 0.002) in the H-V_T_ group compared to the control lambs (Figure 3D).

**FIGURE 4:**
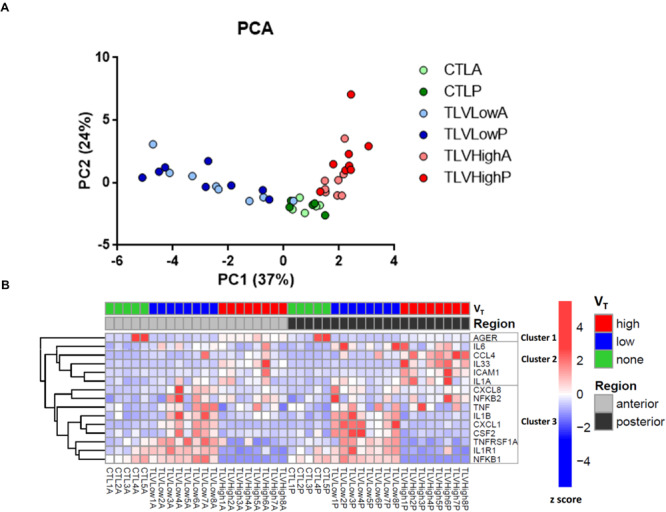
Inflammatory gene expression analyses. **(A)** Principal component analysis of gene expression data in both anterior (Light colored dots) and posterior (Dark colored dots) lung samples from unventilated, L-V_T_ (TLVLow) and H-V_T_ (TLVHigh) lambs. Distance between dots approximates gene-expression pattern differences among experimental conditions/lung regions; the closer the dots, the closer the expression levels for each linearly uncorrelated gene group (i.e., principal component). **(B)** Heatmap and unsupervised clustering of selected gene expression in the anterior and posterior lung regions in L-V_T_, H-V_T_ or unventilated lambs (CTL). Colors indicate expression *z*-score after normalization on housekeeping genes.

A principal component analysis showed clear separation between unventilated, H-V_T_ and L-V_T_ ventilated lamb groups, with consistent gene expression profiles between the anterior and posterior lung regions (Figure 4A). Unsupervised clustering of individual gene expression revealed four main findings: (1) pronounced inflammatory transcriptional changes in both groups of liquid ventilated lungs compared to unventilated lambs; (2) three main gene expression clusters, with an increased pro-inflammatory gene expression profile in L-V_T_ lungs, but not in the H-V_T_ or unventilated lambs; (3) a distinct transcriptional response in lung tissues from L-V_T_ compared to H-V_T_ ventilated lambs; and (4) greater ventilation-associated changes in the posterior, compared to the anterior lung regions (Figure 4B). Altogether, our gene expression analysis indicates that H-V_T_ induces qualitatively distinct transcriptional changes compared to L-V_T_.

## Discussion

Given TLV’s potential to reduce VILI in neonate chronic lung disease, our study provides important information about how to optimize its potential using different levels of tidal volume. The main finding of our study is that TLV with low tidal volume was associated with more inflammatory-gene expression than TLV using high tidal volume. In addition, our results show the possibility to consistently achieve weaning from TLV to spontaneous breathing within 2 h post-TLV.

The overall transcription profile suggests that a TLV strategy using an H-V_T_ may induce less lung inflammation than L-V_T_. Interestingly, observations were consistent between the anterior and posterior regions of the lungs implying that our results are no simply due to a sampling bias. In addition to *IL1B, g*enes encoding for important proteins of the inflammatory cascade, such as *CXCL8*, *TNF*, *CSF2*, and *NFKB1*, were also more expressed in the L-V_T_ group. Nevertheless, *IL33* was upregulated in the H-V_T_ group compared to the L-V_T_ group. This cytokine can be pro-inflammatory or anti-inflammatory depending on its environment (Zhao and Zhao, 2015). In this case, it remains unclear whether the level of *IL33* transcription induced less or more lung inflammation in the H-V_T_ group. Overall, although heterogeneous, the gene-expression profile we found in the L-V_T_ lambs was more consistent with VILI literature data than the profile observed in the H-V_T_ lambs. Moreover, the gene expression profile in H-V_T_ lambs suggests that higher tidal volume may be more lung protective compared to L-V_T_ in TLV. It is interesting to note opposing inflammatory cytokine gene expression profiles between the L-Vt and H-Vt experimental groups. Indeed, instead of having only up regulation in one group and no change in the other, we had genes that were overexpressed in one group while the same genes were down regulated in the other groups. This effect was reproducible in two different regions and therefore is unlikely to represent a measure artifact. This intriguing finding suggests that different tidal volumes trigger qualitatively distinct inflammatory responses in the lungs. These findings definitely require a closer look in a future study, in order to understand the mechanisms involved in these qualitative differences and their clinical implication in terms of choosing the right lung protective ventilatory strategy.

Our results contradict our hypothesis favoring the use of L-V_T_ to prevent lung inflammation. Importantly, L-V_T_ lambs required 50% higher minute ventilation–i.e., a higher respiratory rate–than H-V_T_ lambs in order to maintain PaCO_2_ and pH values within the targeted limits. This was likely due to the known low gas diffusion velocity in perfluorochemicals compared to a gaseous medium (Costantino and Fiore, 2001). This effect could be exacerbated in the ovine model, known for its larger anatomic dead space (Albertine, 2013). It should be noted that our strategy aimed at strictly maintaining the end-expiratory liquid volume at the lowest possible level might have mitigated lung over-distension/volutrauma, a crucial VILI mechanism during TLV, as shown by Kohlhauer et al. (2019). The benefits of limiting the end-inspiratory lung volume might, however, be offset by the disadvantages of the higher respiratory rate required in L-V_T_ lambs. Indeed, a lower respiratory rate has been associated with less alveolar shear stress and lung inflammation during conventional gas ventilation (Hotchkiss et al., 2000; Rich et al., 2000; Vaporidi et al., 2008; Chen et al., 2015).

Very few authors have demonstrated the possibility of achieving complete weaning a neonatal animal after TLV (Jackson et al., 1994; Stavis et al., 1998). Jackson et al. (1994) were able to wean two near-term healthy non-human primates after 5 h of conventional mechanical ventilation following 3 h of TLV, while Stavis et al. (1998) reported weaning three full-term lambs from 4 h of TLV to spontaneous breathing after 16 h. The present study confirms that it is possible to consistently wean healthy full-term lambs from 4 h of TLV to spontaneous breathing within 2 h.

Although the L-V_T_ lambs exhibited a more inflammatory profile, no clinically relevant difference was observed during the weaning process, including oxygen requirement, respiratory rate, and arterial-blood gases. Most animals presented mild respiratory distress during the first 30 to 90 min following extubation. Most required oxygen, and some required continuous positive airway pressure for 4 h after TLV. PaCO_2_ levels and respiratory rate were, however, normal at that point. As anticipated, residual liquid PFC likely affects respiratory mechanics and gas exchange during the first few hours following TLV, while it is being cleared from the lung through evaporation and coughing (Bendel-Stenzel et al., 1998). This is consistent with a past study showing that recovery from TLV in near-term non-human primates was associated with the need for supplemental oxygen in the first 24 to 72 h post-TLV (Jackson et al., 1994). A study is currently underway in our lab to assess respiratory mechanics during weaning after TLV.

### Limitations of the Study

One limitation of our study is its small sample size, which limits power and thus, our ability to demonstrate further differences between the groups. In addition, we decided to proceed to weaning from TLV in order to identify clinically relevant differences post-TLV between the two groups as well as to assess the feasibility of such weaning. The transition from TLV back to gas ventilation might come at the cost of inducing inflammation. Since lambs from both groups were, however, consistently weaned according to the same standardized protocol, we do not believe this introduced a bias in our study. The only animal that could not be extubated because of profound sedation was in the H-V_T_ group; this would have affected the results in favor of the L-V_T_ group, contrarily to our results.

As with all animal models, anatomic and gene expression differences with the human newborn are possible. Clinical trials will be needed before this technology can be used as a standard treatment in the neonatal intensive care units. Gene expression levels were assessed in this study. The authors acknowledge that protein levels as well as functional respiratory assessment after a longer course of TLV would be beneficial in confirming our findings. Nevertheless, this study represents a first step and brings new knowledge in delineating the optimal TLV strategy in newborns.

## Conclusion

Our results suggest that a TLV strategy using high tidal volume and low respiratory rate is associated with reduced lung inflammation compared to low tidal volume and high respiratory rate. TLV modality did not, however, affect weaning to spontaneous breathing, which was achieved within a 4-hour time window. The ability to consistently wean neonatal lambs is a major milestone in TLV research and paves the way for an eventual clinical trial.

## Data Availability Statement

The original contributions presented in the study are publicly available. This data can be found here: https://rnomics.med.usherbrooke.ca/palace?purl=/pcrreactiongroup/list/475.

## Ethics Statement

The animal study was reviewed and approved by Animal Research Ethics Board of the Université de Sherbrooke (protocol #417-17).

## Author Contributions

ÉF-P, PM, J-PP, BC, and MS contributed to the original idea and design of the study. MS, WS, SN, and ÉF-P contributed to the experiments. MS, WS, SN, CMi, ÉF-P, BC, SM, PL, and J-PP contributed to the data collection, analysis, and interpretation. MS, WS, CMo, CMi, SN, SM, PL, BC, J-PP, and ÉF-P all contributed to the manuscript.

## Conflict of Interest

PM and J-PP are co-inventors of patents related to the ventilator prototype used in this study (Apparatus for conducting total liquid ventilation with control of residual volume and ventilation cycle profile, US 7,726,311 B2, EP 1 424 090 B1, CA 2,451,261 and Indirect measurement in a total liquid ventilation system, PCT/CA2014/205548 US 2016/0271348 A1).

The remaining authors declare that the research was conducted in the absence of any commercial or financial relationships that could be construed as a potential conflict of interest.

## Footnotes

1 https://www.r-project.org

## Abbreviations

CPAP: continuous positive airway pressure
CTL: control lambs without ventilation
EELV: end-expiratory liquid volume
EEPP: end-expiratory pause pressure
EIPP: end-inspiratory pause pressure
FgO_2_: fraction of oxygen bubbled in PFOB
FiO_2_: fraction of inspired oxygen
HCO_3_^–^: arterial concentration of bicarbonate ions
H-V _T_: total liquid ventilation with high tidal volume
*IL1B*: interleukin-1 gene
*IL6*: interleukin-6 gene
*IL8*: interleukin-8 gene
L-V _T_: total liquid ventilation with low tidal volume
NAVA: neurally adjusted ventilatory assist
PaCO_2_: partial pressure of carbon dioxide in arterial blood
PaO_2_: partial pressure of oxygen in arterial blood
PEEP: positive end-expiratory pressure
PFC: perfluorochemical
PFOB: perfluorooctyl bromide
PIP: peak inspiratory pressure
SaO_2_: hemoglobin saturation in oxygen (pulse oximetry)
TLV: total liquid ventilation
*TNF*: tumor necrosis factor- α gene
V_*E*_: minute ventilation
VILI: ventilator-induced lung injury
V_T_: tidal volume.
